# Coca

**Published:** 1885-10

**Authors:** 


### Coca.

The coca leaf, when chewed, is a powerful stimulant to the nervous system, of the nature of opium, but less violent and more lasting in its action. Bernays says: "There is so much concurrent testimony as to place beyond doubt the fact that the moderate use of coca leaves as a masticatory enables fatigue to be endured with less distress and with less nourishment." Markham says, that he chewed coca very frequently, and, besides the agreable, soothing feeling produced, he found that he could endure long abstinence from food with less inconvenience thon he would otherwise have felt; and it enabled him to ascend precipitous mountain sides with a feeling of lightness and elasticity, and without losing breath. To the Peruvian Indian Coca is a solace which affords enjoyment, and has a most beneficial effect." Quoting from the same authority: "The incredible fatigue," says Von Tschudi, "endured by the Peruvian infantry, with very spare diet, but with the regular use of coca, and the laborious toils of the Indian miner kept up under similar circumstances throughout a long series of years, certainly afford sufficient ground for attributing to the coca leaves not a quality of mere temporary stimulus, but a powerful nutritive principle."

But the excessive use of Coca is well known to be injurious, and the unsteady gait, the yellow-colored skin, the dim, sunken eyes, the quivering lips and general apathy are the indications of the inveterate coca chewer. It is, however, considered the least injurious of all narcotics in use, and in the higher regions of the Andes its effects are less marked than in warmer and damper districts.

As a palliative agent in the hands of a skillful physician, cocaine is capable of greatly alleviating human suffering, and its use in this manner will henceforth be widely extended.—*Vick's Magazine* for October.

